# *N*^6^-methyladenosine (m^6^A): Revisiting the Old with Focus on New, an *Arabidopsis thaliana* Centered Review

**DOI:** 10.3390/genes9120596

**Published:** 2018-11-30

**Authors:** Susheel Sagar Bhat, Dawid Bielewicz, Artur Jarmolowski, Zofia Szweykowska-Kulinska

**Affiliations:** Department of Gene Expression, Institute of Molecular Biology and Biotechnology, Faculty of Biology, Adam Mickiewicz University, Umultowska 89, 61-614 Poznan, Poland; susbha@amu.edu.pl (S.S.B.); bieda@amu.edu.pl (D.B.)

**Keywords:** *N*^6^-methyladenosine, mRNA m^6^A methyltransferase (MTA), MTB, FIP37, ALKBH, YTDHF, *Arabidopsis*

## Abstract

*N*^6^-methyladenosine (m^6^A) is known to occur in plant and animal messenger RNAs (mRNAs) since the 1970s. However, the scope and function of this modification remained un-explored till very recently. Since the beginning of this decade, owing to major technological breakthroughs, the interest in m^6^A has peaked again. Similar to animal mRNAs, plant mRNAs are also m^6^A methylated, within a specific sequence motif which is conserved across these kingdoms. m^6^A has been found to be pivotal for plant development and necessary for processes ranging from seed germination to floral development. A wide range of proteins involved in methylation of adenosine have been identified alongside proteins that remove or identify m^6^A. This review aims to put together the current knowledge regarding m^6^A in *Arabidopsis thaliana*.

## 1. Introduction

**Figure 1:**
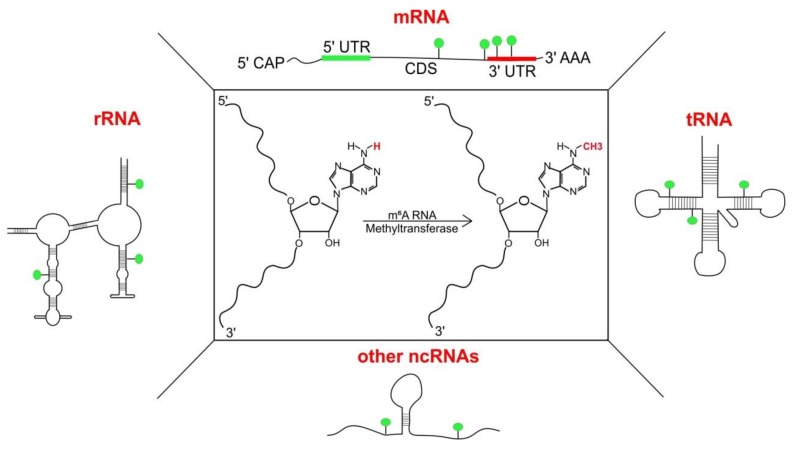
Structure and presence of adenosine methylation at nitrogen-6 position (m^6^A): Substitution of ‘H’ at N6 with ‘CH3’ (central panel, marked in red) in adenosine results in the formation of *N*^6^-methyladenosine. m^6^A has been detected indifferent classes of RNA including messenger RNAs (mRNAs), transfer RNAs (tRNAs) [1], ribosomal RNAs (rRNAs) [2] and other non-coding RNAs (ncRNAs) like small nucleolar RNAs (snoRNAs), long non-coding RNAs (lncRNAs) and primary micro-RNAs (pri-miRNAs).

Methylation of internal adenosine at nitrogen-6 position (m^6^A) is a RNA modification abundant across Eukaryota, and is found in messenger RNAs (mRNAs), transfer RNAs (tRNAs) [1], ribosomal RNAs (rRNAs) [2] and other non-coding RNAs (ncRNAs) [3,4] (Figure 1). m^6^A was first found in mammalian mRNAs in the 1970s [5,6,7] followed by plants [8,9] and viruses [10,11]; later in yeast [12] and much more recently in bacteria [13]. With its discovery, m^6^A was added to a pool of other RNA modifications [14,15] but its role remained largely obscured till the late 1990s. m^6^A was challenging to study, as it does not cause any changes in Watson–Crick base pairing and thus could not be detected by traditional reverse transcription methods [16]. With the technological advances surrounding scientific techniques and new methods being developed for functional analysis of such RNA modifications, m^6^A became relevant again, surprisingly, with the discovery that this modification is reversible [17] and has an influence on a variety of essential processes in cells [18].

First reports of m^6^A in plant mRNAs date back to 1979, when it was first found in wheat (*Triticum aestivum*) and maize (*Zea mays*) [8,9]. Soon after, m^6^A mark in mRNA was reported in oat (*Avena sativa*) [19]. Interestingly, shortly after the first discovery of m^6^A in mRNAs of plants, scientists were able to identify the sequence motif RR**A**CH (where R = G/A, H = A/C/U, bold letter stands for adenosine being modified to m^6^A) which was in consensus with the animal sequence motif [20]. After these initial discoveries, the interest in plant m^6^A gradually decreased and was regained after its discovery in *Arabidopsis thaliana* which was reported much later in 2008 by Zhong and colleagues [21]. It was also found, that although the sequence motif in animals and plants is conserved, the sites of m^6^A enrichment show some differences between them: While m^6^A in animals is highly enriched near the 3′UTRs and stop codons [3,22], *Arabidopsis* m^6^A mRNA methylome also shows enrichment near the start codon [23] besides 3′UTRs and stop codons.

## 2. m^6^A: The Methyltransferase Complex

The fact that mRNA m^6^A methyltransferase complex in animals is a multi-protein complex [24] was known for a long time. Using nuclear extracts from HeLa cells, Bokar and colleagues pulled down the m^6^A methyltransferase complex (which was named as m^6^A-MT) using a synthetic RNA with the known binding site as a bait. They identified three major components of the complex and designated them as MT-A1 (30 KDa), MT-A2 (200 KDa) and MT-B (875 KDa). It was also noted that all three components had to be present to observe any m^6^A methylation activity. Later, Bokar and colleagues also identified a 70 kDa protein from MT-A2 subcomplex as containing the *S*-adenosylmethionine (AdoMet) binding domain and it was named as MT-A70/METTL3 [25].

The characterization of *Arabidopsis* mRNA m^6^A methyltransferase complex was initiated by the identification of a homolog of human mRNA m^6^A methyltransferase (MTA-70/METTL3): mRNA adenosine methylase (MTA) [21]. The functional importance of MTA was also demonstrated by Zhong and colleagues by loss of function studies, where disruption of the *MTA* gene led to embryo lethality. This observation is in consensus with results from studies on animals, as disruption of MTA/METTL3 homologs has been shown to be embryo lethal in mice [26], causes apoptosis in mammalian cells [27] and leads to increased female lethality in *Drosophila melanogaster* [28,29]. To overcome the obvious difficulties in studying the role of m^6^A or lack thereof in *Arabidopsis*, Bodi and colleagues complemented the KO mutant of *MTA* with *ABI3:MTA*. The *ABI3* promoter drives the expression of *MTA* in the embryonic stages of development, enough to rescue the embryo-lethal phenotype, and at a vastly reduced level at later stages [30]. Expression analysis of MTA also showed that it is mostly expressed in dividing tissue (apical meristems, reproductive organs and seeds) while the expression levels are quite low in other tissues; m^6^A methylation levels also vary in the tissues accordingly [21].

While the characterization of *Arabidopsis* MTA has been performed and shown in detail, its protein partner, *Arabidopsis* MTB, has been characterized less intensively. MTB is the homolog of human METTL14, which has also been shown to be a part of m^6^A methyltransferase complex [31]. On the lines of homology with the human METTL14, MTB was considered to be a possible m^6^A methyltransferase complex partner [32], but surprisingly no study focusing solely on MTB and its role on m^6^A methylation can be found. MTB has been characterized as embryo defective and complete lack of MTB is also embryo lethal. A 2017 study, focusing on newer components of m^6^A methyltransferase complex (discussed below) also takes MTB into account [33]. In their study, Růžička and colleagues, found that MTB associated with MTA and also formed homodimers. MTB was also shown to interact with the newly discovered components of m^6^A methyltransferase complex as identified in the same study. Experiments on RNA interference RNAi lines with inducible knockdown of *MTB* show that knockdown of *MTB* leads to nearly a 50% reduction in m^6^A levels [33]. 

The first characterization of an MTA protein partner in the mRNA m^6^A methyltransferase complex was reported from *Arabidopsis*, in the form of FKBP12 interacting protein 37 kDa (*At*FIP37) [21,34]. FIP37 is a homolog of human WTAP (Wilms’ Tumour1-Associating Protein) and *Drosophila* FL(2)D (Female Lethal2) [21]; disruption of *AtFIP37* is also embryo lethal [35]. Using an approach identical to Bodi et al., transgenic lines with *LEC1:FIP37* (embryo specific) and also *ABI3:FIP37* constructs were used by researchers to show that low levels of FIP37 cause developmental defects in the shoot apical meristem (SAM), without proper leaf production and eventual death of plants. *FIP37* expression patterns are similar to that of *MTA*’s, and as one would expect, m^6^A levels in plants with low levels of FIP37 were found to be much lower than in wild type (WT) plants [34].

**Figure 2:**
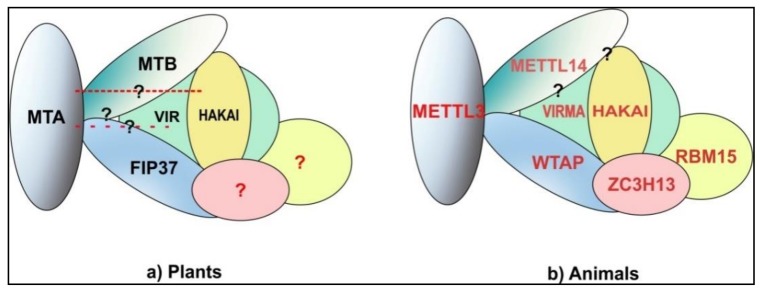
m^6^A methyltransferase complex: (**a**) PlantsMTA-MTB-FIP37 form the core components of the *Arabidopsis* m^6^A methyltransferase complexof which VIR and HAKAI are also a part.VIR and HAKAI interact with MTB and FIP37 but no interaction with MTA has been shown yet. Similarly, direct interactions between MTB and FIP37 have not been shown yet. Whether homologs of animal ZC3H13 and RBM15 are a part of plant methyltransferase complex still needs to be shown. The components of methyltransferase complex here are shown on the basis of data from studies on *Arabidopsis*. (**b**) Animal interactions between all three core components METTL3-WTAP-METTL14 has been shown. VIRMA (KIAA1429) (VIR homolog) and HAKAI have been shown to be part of the complex via interactions with WTAP and their interaction with METTL14 is yet to be elucidated. Most recent data suggests that WTAP along with RBM15/ZC3H13/HAKAI/VIRMA provides a scaffold for METTL3/METTL14 for methylation [39,40,41,42]. The animal model is based on data from mammals and *Drosophila*. Question marks depict possible but not yet identified interactions.

While MTA, MTB and FIP37 are largely considered to form the core of m^6^A methyltransferase complex, they definitely are not the only proteins involved in m^6^A methylation. Růžička and colleagues showed that a homolog of human VIRMA (KIAA1429) (*Drosophila*—Virilizer (VIR)) [36,37] is also a part of *Arabidopsis* m^6^A methyltransferase complex, along with the homolog of human HAKAI (an E3 Ubiquitin ligase) [33,38]. VIR is also essential for embryo development and null alleles are embryo lethal. Furthermore, the authors also show significant reductions of m^6^A levels in *vir* and *hakai* mutants (where both proteins are expressed inefficiently). Interestingly, while the lack of all other proteins discussed above result in similar sever phenotypes, *HAKAI* null mutants are not only viable but also show phenotypes similar to WT plants while some phenotypic defects are still present [33]. While the association of these new members with the m^6^A methyltransferase complex and their necessity for m^6^A methylation has been proven, they have not yet been attributed to any specific role within the complex. Figure 2 shows the comparison of m^6^A methyltransferase complexes in plants and animals.

## 3. m^6^A: Readers and Erasers

It is known that methylation of adenosine does not alter nucleotide base pairing, but it alters the secondary structure of RNA and plays a role in RNA-protein interactions [43,44,45,46,47]. The change in RNA secondary structure is mainly brought about by lowered thermodynamic stability of RNA duplexes containing the m^6^A mark [43]. It was also shown that the presence of m^6^A in a hairpin loop leaves the double stranded region more accessible to various proteins, a phenomenon that is termed as “m^6^A switch” [44,45,46]. It was also shown that the presence of m^6^A in hairpin loop destabilizes the double stranded structure, while single stranded RNA (ssRNA) is stabilized by m^6^A and presence of m^6^A causes the adjacent area to resemble single stranded RNA, thus leaving it exposed to RNA binding proteins [47]. Lastly, proteins that directly identify the m^6^A mark have also been identified. YT521-B homology (YTH) domain family proteins (YTHDF) is an example of such a family of proteins that has been shown to recognize m^6^A and bind RNA [48,49,50,51,52]. Thus, it is not hard to imagine that a majority of biological effects of reduced m^6^A levels are a result of RNA secondary structure distortions and/or lack of interactions between certain proteins identifying m^6^A marks in mRNAs and facilitating further processes in plants. These proteins, that can identify m^6^A, are called “reader” proteins. On the other hand, proteins (demethylases) that identify m^6^A and affect RNA metabolism by removing m^6^A are called “erasers.” Recently, researchers working on plants have focused on readers and erasers to explain the physiological effects that accompany the lack of m^6^A. 

In plants, 13 YTH family proteins are known to occur and out of those 11 have been identified as Evolutionarily Conserved C-Terminal Region proteins 1–11 (ECT 1–11). The first report of two ECT proteins (ECT1/2) in 2005 by Ok and colleagues associated them with calcium signaling and showed that the conserved C-terminal domain (YTH domain) is necessary for their nuclear localization. ECT2 has since been shown to bind m^6^A in *Arabidopsis* [48,50,51,52]. Scutenaire and colleagues show that ECT2 binds to m^6^A via a tri-tryptophan pocket, and if these amino acids are mutated, ECT2 loses its m^6^A binding ability. They also show that *ect* mutants share phenotypes (defective trichomes) with *mta* mutants and FIP37 overexpressing transgenic lines. They also show that the altered trichome morphology is a result of higher cell ploidy caused by endoreduplication [50]. On the other side, Wei and colleagues demonstrate that ECT2 binds to m^6^A at a motif, URUAY (R=G>A, Y=U>A), that is not similar to the MTA methylation motif of RRACH. They present URUAY as a new plant specific motif that plant m^6^A methyltransferase can recognize and methylate [51] and this data is supported by recently published data from another group who also found enrichment of U rich motif in their analysis regarding global mapping of uncapped and cleaved transcripts [53]. Providing a more molecular mechanism for the altered trichome morphology by ECT2, Wei and colleagues show that ECT2 improves the stability of m^6^A methylated RNAs transcribed from genes involved in trichome morphogenesis. This observation is opposite to the reported decrease in stability of RNAs caused by YTHDF proteins’ binding this mark in animal system [54]. While animal YTH domain protein (YTHDF2) causes accelerated deadenylation of transcripts carrying the m^6^A mark, Anderson and group show that, in plants, m^6^A prevents ribonucleolytic cleavage of such transcripts (as observed by Wei and colleagues too) [51,53,54]. In a study focused more on the morphological aspects of ECT proteins, including ECT2/3 and4, Arribas-Hernández and colleagues show that these proteins are intrinsically important for proper leaf morphogenesis including trichome branching [52]. These results provide answers to the observations made in the very beginning of MTA characterization regarding trichome branching [30].

Similar to the “reader” proteins, “erasers” can also modulate various biological processes that result in certain distinct phenotypes and hence help in identification of newer pathways where m^6^A plays a role. AlkB family of non-heme Fe(II)/α-ketoglutarate (α-KG)-dependent dioxygenases family proteins and their homologs (ALKBH), are known to act as m^6^A demethylases in mammalian systems [17,55]. ALKBH family proteins are also found in *Arabidopsis* and two of them, ALKBH9B and ALKBH10B, have been shown to be active m^6^A demethylases concerning plant systems [56,57]. ALKBH9B was the first m^6^A demethylase reported from the *Arabidopsis* system, although it is shown to affect viral ssRNA methylation status. With the spotlight on alfalfa mosaic virus (AMV), researchers demonstrate that ALKBH9B positively affects viral abundance in plant cells [57]. Working on this further, the group also found that ALKBH9B can demethylate m^6^A from ssRNA in vitro. They also show that the viral RNA gets m^6^A methylated upon infection. Depletion of ALKBH9B led to the viral RNA being hypermethylated and eventually degraded by Non-sense Mediated Decay (NMD). These observations, when combined with the fact that lack of ALKBH9B led to lower infection levels, allowed the authors to conclude that methylation status plays a vital role in modulating viral infections in *Arabidopsis* [57].

Interestingly, a study done in *Nicotiana tobaccum* also co-relates m^6^A with viral infections [58]. Li and colleagues show that upon infection with tobacco mosaic virus (TMV), methylation levels were decreased in *Nicotiana* plants while simultaneously the expression level of a protein homologous to human ALKBH5 demethylase went up [58]. This indicates that a mechanism similar to *Arabidopsis* exists in other plants as well.

ALKBH9B, while interesting as m^6^A demethylase, was not demonstrated to work on any RNA substrate endogenous to *Arabidopsis*. In order to focus on demethylase that would be more involved in plant metabolism, Duan and colleagues targeted another ALKBH family protein, ALKBH10B. ALKBH10B binds to and demethylates m^6^A RNA both in vitro and in vivo. Scientists observed that disruption of ALKBH10B leads to global upregulation of mRNA methylation. This upregulation could be seen in genes that are involved in several developmental pathways and organ development among others. One of the phenotypic effects of ALKBH10B over/under expression observed by the authors was altered flowering time. *alkbh10b* null mutants flower later than WT plants and ALKBH10B overexpressing plants flower earlier when compared to wild type plants. This phenomenon was attributed to the methylation status of mRNAs corresponding to several genes involved in proper flowering, especially but not limited to, Flowering Locus T (*FT*) [56]. Researchers were able to show that ALKBH10B directly binds to *FT* mRNA (which contains m^6^A residues) and demethylates it. In *alkbh10b* background, *FT* transcripts are degraded faster as compared to WT plants, linking m^6^A levels to the degradation of mRNA.

## 4. m^6^A: Physiological Roles

The fact that the lack of m^6^A methylation (or any of the core mRNA m^6^A methyltransferase complex proteins) is lethal for plants highlights the importance of this modification in primary metabolism of plants. m^6^A methylation in *Arabidopsis* could be associated with a variety of stress and/or stimuli responses, as was indicated by the analysis of gene expression comparing WT and *mta* mutants [23,30]. m^6^A marks have been found in mRNAs of photosynthesis related proteins, notably Serine/Threonine Protein Kinase (STN8), indicating towards some role of m^6^A in this vital process [23]. Upon investigation of m^6^A levels in different organs, it was observed that m^6^A methylation levels, varied in different organs indicating towards some role of m^6^A in organ development [21,59]. Zhong and colleagues show that m^6^A abundance is the highest in young seedlings and flower buds followed by leaves and roots. Contrary to this, Wan and colleagues reported that methylation levels were the highest in leaves, followed by flowers and roots. They also report that transcripts with higher levels of methylation in each organ are different and somehow co-related. For example: transcripts related to photosynthesis were methylated at a higher level in leaves as compared to flowers or roots; transcripts related to alkaloid biosynthesis had higher methylation levels in roots as compared to the other two organs etc. In the same study, Wan and colleagues, also report presence of the m^6^A mark in mRNA of various transporter and signal transduction proteins. They also observed that methylation levels were generally higher in transcripts that are normally present at lower levels in the cell. The authors speculate that m^6^A methylation may play a role in providing stability to such low expressed transcripts [59]. m^6^A methylation patterns were also found to be strain specific, as Luo et al. found that two accessions (Can-0 and Hen-16) had strain specific enrichment of the m^6^A mark. The enrichment of m^6^A in these two strains also co-related to the different gene expression levels pointing towards the differences in m^6^A patterns to different geographical habitat of these strains [23]. Recently, studies to understand the role m^6^A in plants other than *Arabidopsis* like tobacco (discussed earlier) and rice have also been undertaken [60]. Genome wide identification of rice m^6^A transcripts in two different tissue types (differentiated callus and leaves) revealed an organ specific m^6^A pattern in transcripts (as observed previously in *Arabidopsis*); and a negative correlation between m^6^A methylation enrichment and gene expression [60].

The presence or lack of m^6^A marks in transcripts is not a definitive sign that it plays some significant role in plant metabolism. A more physiologically relevant proof of such role comes from experiments that show the physiological effects of reduced m^6^A levels. When MTA level is lower, plants show developmental defects like reduced inflorescence length, increased trichome branching with crinkled leaves and differences in flower morphology with reduced seed production [30]. Increased trichome branching is also observed in *Arabidopsis* line over-expressing FIP37 protein [35]. A pioneering study that related m^6^A levels directly to morphological changes, focused on FIP37 [34]. Shen and colleagues found that the deficiency of FIP37 caused overproliferation of SAM tissue. After showing that FIP37 is otherwise highly expressed in dividing tissue and it is necessary for m^6^A methylation, the authors found two genes (*WUSCHEL (WUS)* and *SHOOTMERISTEMLESS (STM)*) involved in SAM proliferation that have an m^6^A mark in their mRNAs. Upon depletion of FIP37, the depletion of m^6^A was accompanied by over accumulation of these two mRNAs. This overaccumulation was shown to be a result of slower degradation of these mRNAs because of the lack of m^6^A marks. The authors also demonstrated the overaccumulation of these transcripts and their lack of methylation when MTA levels were reduced, using artificial microRNA (miRNA) based RNAi technology [34]. Through their experiments Shen and colleagues clearly demonstrated a pathway where the m^6^A mark enhances degradation of certain mRNAs and plays a significant role in their homeostasis which is reflected in proper plant growth and development.

Recent work by Anderson and colleagues, shows that m^6^A stabilizes transcripts as it protects them from ribonucleolytic cleavage [53]. An increase in cleavage of transcripts, 4–5 nt upstream of adenosine, in the absence of m^6^A proved that m^6^A indeed protects these transcripts from cleavage. Moreover, under stress, m^6^A also stabilizes stress responsive gene transcripts [53]. As discussed in earlier sections, m^6^A is also associated with viral infections (biotic stress) and its presence in a variety of transcripts could link this modification to many more vital processes.

**Figure 3:**
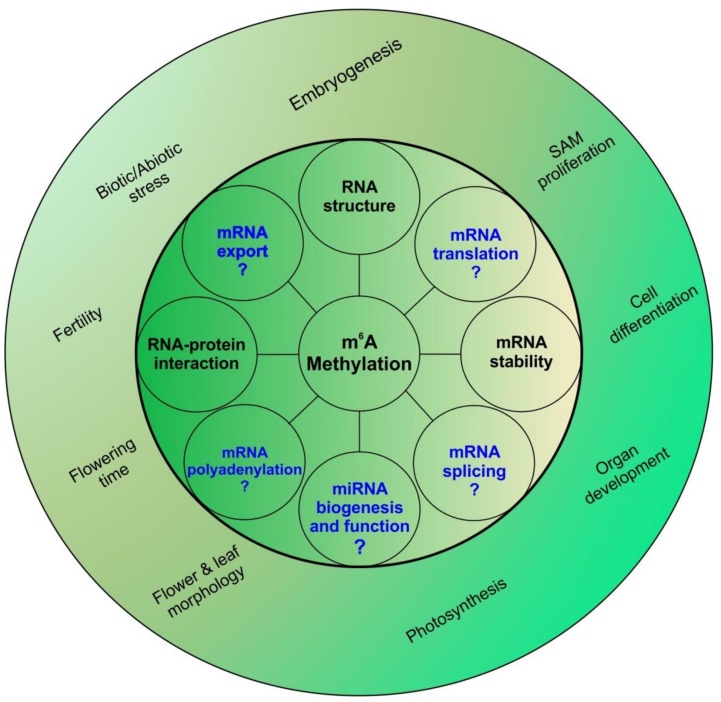
m^6^A and its many roles: m^6^A methylation has been known to alter mRNA stability and structure along with altering RNA-protein interactions at the molecular level. These changes in molecular levels are translated to a large array of physiological changes ranging from photosynthesis to stress response. In animal systems, m^6^A has also been shown to affect mRNA splicing, export, polyadenylation and translation; whether these also play a role in plants remains to be understood. m^6^A has been shown to be vital for proper shoot apical meristem (SAM) proliferation and organ development. Flower growth, morphology and fertility are also affected by m^6^A. In animals m^6^A has also been associated with microRNA (miRNA) biogenesis; whether this is true for plants is still unknown.

Apart from affecting mRNA metabolism, some other roles of m^6^A have already been elucidated in animal systems; one such role is in miRNA biogenesis. In 2015, researchers showed that m^6^A methylation acts as a mark for further processing of mammalian miRNAs [61]. Developing on this further, it was also shown that Heterogenous Nuclear Ribonuclear Protein A2B1 (HNRNPA2B1) identifies the m^6^A mark and facilitates further processing of pri-miRNAs [62]. To our knowledge, no information regarding the role of m^6^A marks in plant miRNA biogenesis is available. Figure 3 summarizes physiological and molecular roles of m^6^A in plants. 

## 5. Conclusions and Future Perspectives

While the field of m^6^A in plants is still evolving, it is clear that m^6^A methylation plays a definitive role in plant development. In animal systems, m^6^A has been shown to play a role in processes ranging from mRNA stability, splicing, maturation to cell differentiation and stress response (for a review: Reference [14]). In close agreement, m^6^A in plant systems also plays roles similar to animal counterparts, as discussed in this review. While we know that m^6^A alters mRNA stability in *Arabidopsis*, it appears that these changes are not universal. As discussed earlier, Anderson and colleagues [53] prove that m^6^A methylation provides stability to mRNAs while Shen and colleagues [34] demonstrate that it promotes transcript degradation. Contradictory results were also reported regarding the extent of m^6^A methylation between organs, case in point: Two studies by Zhong and colleagues [21], and Wan and colleagues [59]. Zhong and colleagues used TLC to detect m^6^A and report that flower buds contain the most amount of m^6^A, while based on m^6^A sequencing, Wan and colleagues report that m^6^A methylation is highest in leaves. While highest methylation levels in flowers would be rational, considering that MTA is highly expressed in actively dividing tissue, higher m^6^A levels in leaves was explained by Wan and colleagues as a consequence of higher photosynthetic and metabolic importance of leaves. Accordingly, we should keep in mind that this could be a matter of different sensitivities of the two different techniques used in the two studies and as the field is still incorporating new tools for analysis, such discrepancies might arise from time to time. On the other hand, the data generated so far clearly suggests that m^6^A might influence cell metabolism in very complex patterns. Recent data also points to some possible differences between the animal and plant sequence motif within which m^6^A is deposited. As discussed earlier, UGAU has been reported as a possible m^6^A methylation motif in *Arabidopsis* by two different groups [51,53]. Furthermore, data shows that m^6^A methylation is enriched in some organs of plants than others, and apart from the information that the methylation of transcripts correspond to their function and importance in the organs, a lot needs to be known about the underlying mechanisms regarding such regulation. This is also the case of difference in m^6^A enrichment across *Arabidopsis* strains and could point that m^6^A is also affected by the environmental cues that are not necessarily stress related. Lastly, a possibility that m^6^A levels in a particular organ in one plant could also vary with time (day or night) cannot be overlooked. As mentioned earlier, m^6^A also plays a role in miRNA biogenesis in mammals. miRNAs are known to be important players in plant stress responses [63,64,65] and many stress responsive gene transcripts have been found to be m^6^A methylated. Keeping that in mind, it would be interesting to see whether miRNA biogenesis influenced by m^6^A works in conjunction with m^6^A methylation of mRNAs to modulate stress responses. Characterization of more downstream proteins i.e., the “readers” and “erasers”, will further our understanding of the numerous ways m^6^A may affect plant development. 

For an epigenetic modification that has been known since the 1970s, relevant techniques and instruments necessary to study m^6^A did not develop at the same pace, thus m^6^A remained relatively unexplored by researchers. Knowing that m^6^A modification is essential for life and thanks to the rapid advancement of technology and gradual increments in our understanding of this abundant and reversible modification, the future for m^6^A is filled with many more important questions that need to be answered.
